# Tunable superconductivity and its origin at KTaO_3_ interfaces

**DOI:** 10.1038/s41467-023-36309-2

**Published:** 2023-02-20

**Authors:** Changjiang Liu, Xianjing Zhou, Deshun Hong, Brandon Fisher, Hong Zheng, John Pearson, Jidong Samuel Jiang, Dafei Jin, Michael R. Norman, Anand Bhattacharya

**Affiliations:** 1 Materials Science Division, Argonne National Laboratory, Lemont, IL 60439 USA; 2 Department of Physics, University at Buffalo, SUNY, Buffalo, NY 14260 USA; 3 Center for Nanoscale Materials, Argonne National Laboratory, Lemont, IL 60439 USA

**Keywords:** Superconducting properties and materials, Surfaces, interfaces and thin films, Electronic properties and materials

## Abstract

What causes Cooper pairs to form in unconventional superconductors is often elusive because experimental signatures that connect to a specific pairing mechanism are rare. Here, we observe distinct dependences of the superconducting transition temperature *T*_c_ on carrier density *n*_2D_ for electron gases formed at KTaO_3_ (111), (001) and (110) interfaces. For the (111) interface, a remarkable linear dependence of *T*_c_ on *n*_2D_ is observed over a range of nearly one order of magnitude. Further, our study of the dependence of superconductivity on gate electric fields reveals the role of the interface in mediating superconductivity. We find that the extreme sensitivity of superconductivity to crystallographic orientation can be explained by pairing via inter-orbital interactions induced by an inversion-breaking transverse optical phonon and quantum confinement. This mechanism is also consistent with the dependence of *T*_c_ on *n*_2D_. Our study may shed light on the pairing mechanism in other superconducting quantum paraelectrics.

## Abstract (web summary)

A distinct dependence of the superconducting transition temperature on carrier density for electron gases formed at KTaO_3_ interfaces is reported. In addition, these interfaces are shown to play a role in mediating superconductivity in this system. The crystallographic orientation and carrier density dependent superconductivity at KTaO_3_ interfaces can be explained by Cooper pairing via inter-orbital interactions and quantum confinement.

## Introduction

Ever since the advent of superconductors, investigations of the mechanism of superconductivity have been at the forefront of condensed matter physics^1–8^. Recent years have seen the discovery of superconductivity in a plethora of engineered material systems, such as^9,10^ LaAlO_3_/SrTiO_3_, FeSe/SrTiO_3_ and twisted bilayer^11^ and trilayer^12,13^ graphene. These interfacial superconductors are of great interest due to the unconventional nature of their superconductivity, offering new routes towards pairing of electrons, as well as the tunability of their properties using electric field-effect gating^14^. For example, the presence of spin-orbit coupling and broken inversion symmetry at interfaces could enable new channels for Cooper pairing^15^, and for realizing a pairing state^15,16^ that can host zero-modes with non-Abelian statistics^17,18^. Insights into the pairing mechanisms in these material systems are thus of great interest, and an area of intense experimental and theoretical research.

In this work, we investigate the origins of superconductivity discovered at oxide-insulator/KTaO_3_ interfaces^19^ through chemical doping and electric field gating. We establish the doping dependence of *T*_c_ for KTaO_3_ (KTO) interfaces of different crystallographic orientations. A remarkable proportionality where *T*_c_ ∝ *n*_2D_ over nearly an order of magnitude is observed at the EuO/KTO (111) interface. In contrast, no superconductivity is observed at the (001) interface of KTO down to 25 mK for all *n*_2D_, while the KTO (110) interface superconducts at a *T*_c_ as high as 1 K, intermediate between the (111) and (001) cases. Using electric field-effect gating, we tune both *n*_2D_, as well as the confinement of electrons to the interface. We find that *T*_c_ increases for both. We rule out the possibility that *T*_c_ is controlled by the phase stiffness temperature^20^. We then discuss possible scenarios for pairing and find that inter-orbital interactions mediated by the soft transverse optical phonon reproduces key findings in our measurements, particularly the extreme sensitivity of *T*_c_ to the crystallographic orientation of the interface, and the consistency with the linear dependence of *T*_c_ with *n*_2D_.

## Results

### Distinct *n*_2D_ dependences of *T*_c_ at KTO (111), (001), and (110) interfaces

Two-dimensional electron gases (2DEGs) at KTO interfaces are obtained by growing an oxide overlayer EuO on (111), (001), and (110) KTO surfaces using molecular beam epitaxy (MBE). The charge carriers at the EuO/KTO interface originate from chemical doping of oxygen vacancies and/or Eu substitution for K near the interface with KTO^19^. The carrier density *n*_2D_ in these samples is determined using Hall measurements at 10 K. *n*_2D_ of the as-grown samples can be tuned by nearly a factor of 10 by varying the growth conditions (see Methods section). We find that the donor state of charge carriers in these samples evolves gradually from shallow to deeper energy as *n*_2D_ increases, the details of which are presented in the Methods section and Supplementary Fig. 1.

**Fig. 1:**
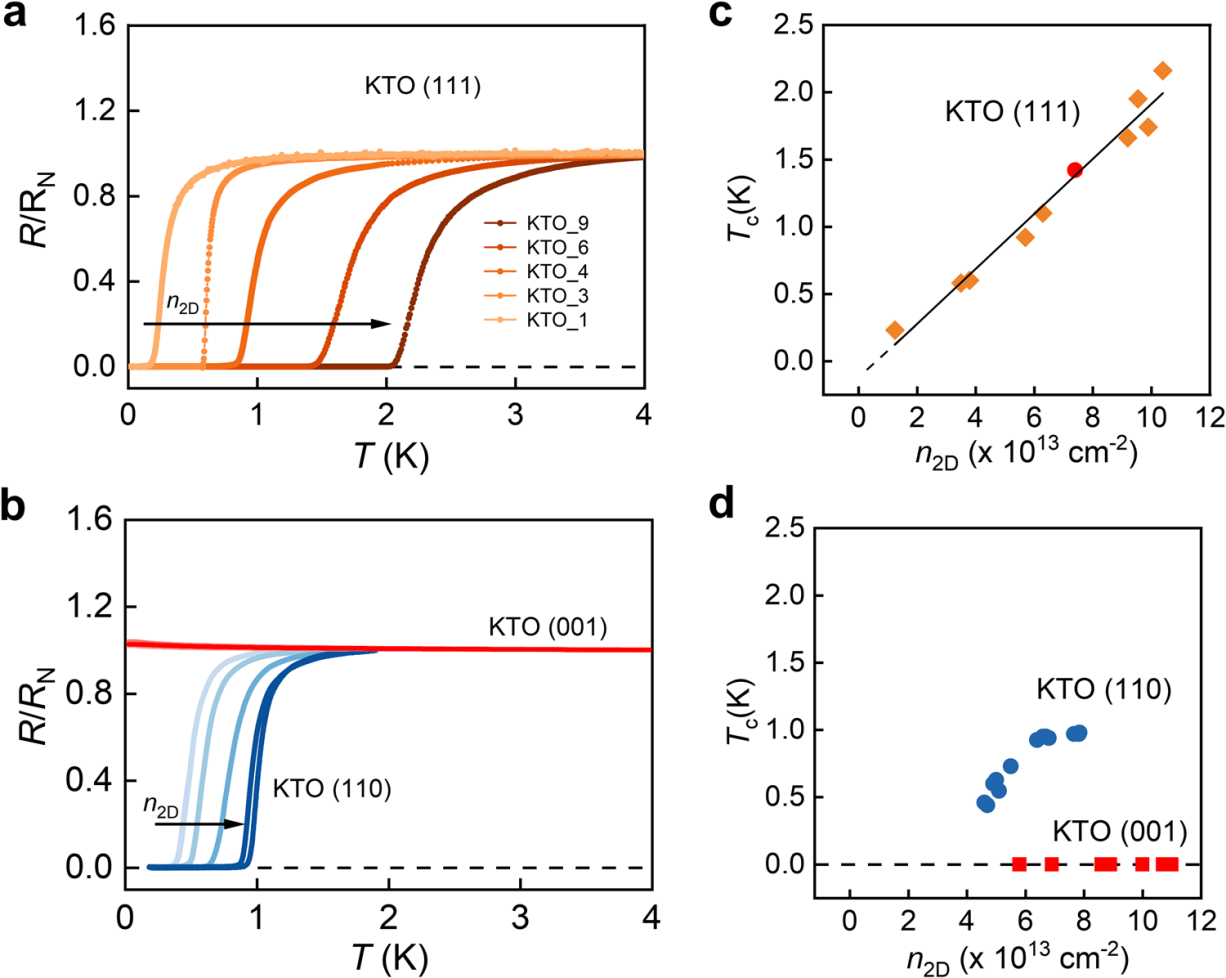
*T*_c_ tuned by *n*_2D_ through chemical doping. **a**, **b** The ratio of the resistance *R*_s_/*R*_N_ as a function of temperature measured for KTO (111) samples (**a**) and KTO (001), (110) samples (**b**), respectively, with varying *n*_2D_. The direction of the arrow indicates the increase of *n*_2D_. **c**, **d** *n*_2D_ dependence of *T*_c_ for KTO (111) samples shown in (**a**) and KTO (001), (110) samples shown in (**b**), respectively. The red circle data point in (**c**) is taken from Ref. ^59^.

The EuO/KTO(111) samples are labeled as KTO_#, with # being in the order of increasing *n*_2D_. Figure 1a shows the temperature dependence of the ratio of the sheet resistance *R*_s_/*R*_N_ (*R*_N_ is the normal-state resistance at 4.5 K) for KTO (111) samples with different *n*_2D_. The superconducting transition defined by *R*_s_/*R*_N_ going to zero occurs at progressively higher temperatures with increasing *n*_2D_. We note that the variation in the sheet resistance in the normal state in these samples produces differences in the superconducting transition width. This behavior is expected for a 2D system when the conductance is enhanced by superconducting fluctuations. Figure. 1b shows the measurement of *R*_s_(*T*) on EuO/KTO (001) and (110) samples. As reported before^19^, no superconducting transition can be measured for the (001) samples down to the lowest temperatures of ~25 mK. On EuO/KTO(110) samples, we observe that superconductivity emerges below about 1 K, which is similar to that reported for the LaAlO_3_/KTO (110) interface^21^. The transition temperature *T*_c_ for the KTO (110) samples also increases with *n*_2D_, but gradually saturates.

When *T*_c_ (determined at 20% of *R*_N_) of all the KTO (111) samples are plotted against *n*_2D_, as shown in Fig. 1c, we observe a largely linear dependence of *T*_c_ on *n*_2D_. The solid line is a fit using *T*_c_ = *a*· *n*_2D_ + *b*, where the coefficient *a* ≈ 0.2 × 10^−13^ K cm^2^, and the *y*-axis intercept *b* ≈ −0.1 K. We note that the fitting line extrapolates to near the origin of the axes, which implies that the undoped KTO (111) interface is near a doping induced critical point separating a band insulator and a superconducting phase. The linearity shown here is insensitive to the choice of *T*_c_ as shown in Supplementary Fig. 1.

Within the entire doping range, *T*_c_ versus *n*_2D_ does not show a dome, suggesting that *T*_c_ may be further enhanced by increasing the doping level. In Fig. 1d we plot *T*_c_ versus *n*_2D_ for KTO (001) and (110) interfaces, which show entirely different behavior than that of the KTO (111) interface: *T*_c_ cannot be measured, down to 25 mK, for all *n*_2D_ for the KTO (001) interface; while *T*_c_ for the KTO (110) interface increases with *n*_2D_ but saturates at a value about half that of the (111) case. We have also measured the Ginzburg-Landau coherence length (ξ_GL_) in the KTO (111) samples with different *n*_2D_, and found that ξ_GL_ satisfies a scaling relation between *T*_c_ and Hall mobility. The details are presented in the Methods section and Supplementary Fig. 2.

### Electrostatic tuning of superconductivity in a low *n*_2D_ sample

**Fig. 2:**
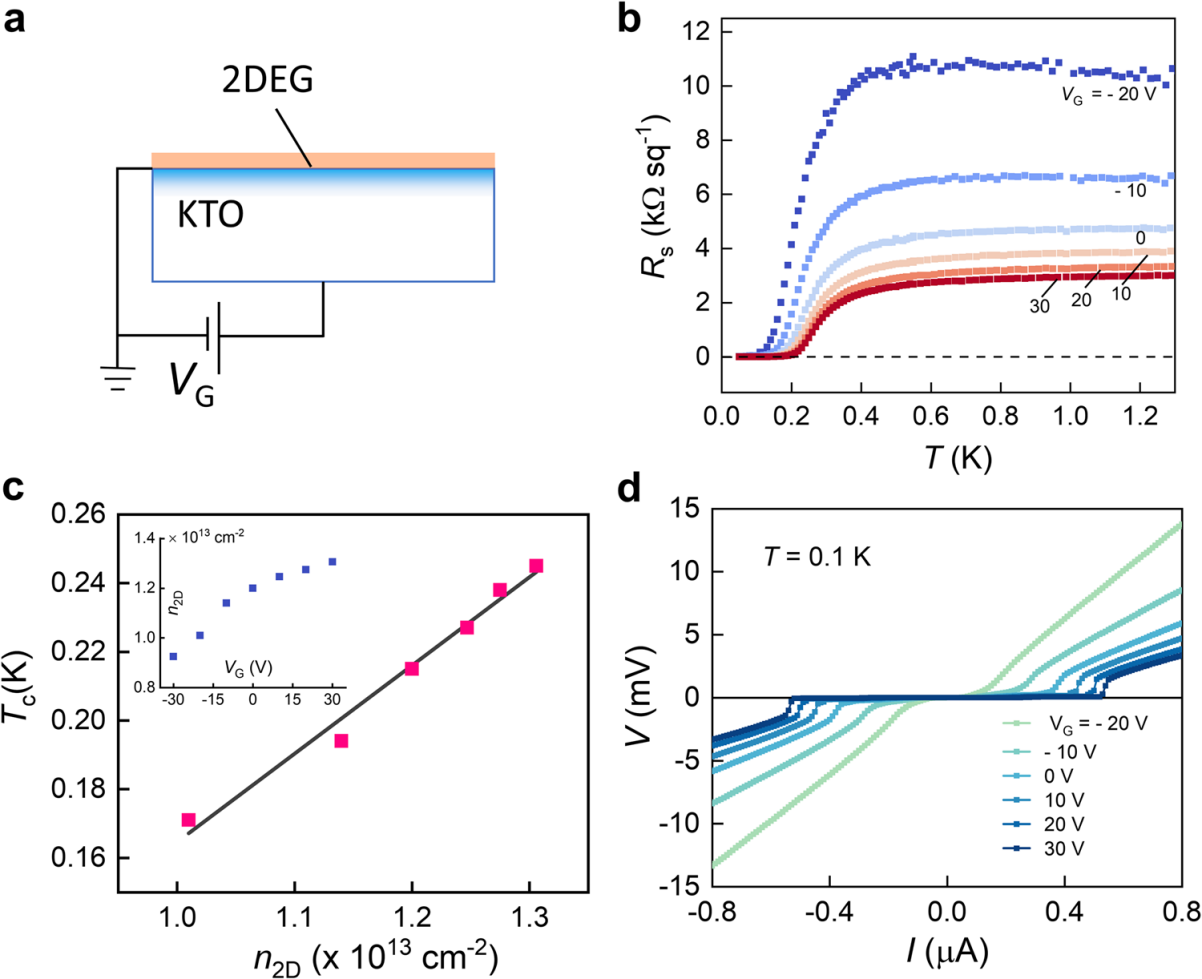
Electrostatic gating on low *n*_2D_ sample. **a** Schematic of the back gate measurement geometry. **b** *R*_s_ versus *T* measurements on KTO_1 for different *V*_G_. **c** *T*_c_ versus *n*_2D_ controlled by electrostatic gating. Inset shows *n*_2D_ as a function of *V*_G_. **d** *V*–*I* measurement for different *V*_G_ at *T* = 0.1 K.

To gain further insight into the mechanism of the superconductivity, we have performed electrostatic gating measurements on the EuO/KTO (111) samples, in both the low and high *n*_2D_ limits. It is expected that electric fields can tune a relatively large fraction of charge density in the low *n*_2D_ sample in a back-gate geometry, given the large dielectric constant (~4500) of KTO at low temperatures^22–24^. Figure 2a shows a schematic of our measurement. A 100-nm thick Pt film deposited on the bottom side of KTO is used as the positive electrode, while the 2DEG at the EuO/KTO interface is grounded.

Figure 2b shows the measurements of *R*_s_(*T*) for different gate voltages *V*_G_ on KTO_1, which has the lowest carrier density *n*_2D_ = 1.25 × 10^13^ cm^−2^. For this sample, *R*_N_ increases by over three times when *V*_G_ is varied from 30 V to –20 V. Correspondingly, *T*_c_ becomes lower. We have measured *n*_2D_, which increases with *V*_G_ monotonically, as shown in the inset of Fig. 2c. By using a parallel-plate capacitor approximation and the thickness of KTO substrate of 0.5 mm, the slope of *n*_2D_ vs. *V*_G_ at *V*_G_ = 0 V yields a dielectric constant for KTO of ~5700, which is close to the known value for bulk KTO. When *T*_c_ is plotted against *n*_2D_ as tuned by *V*_G_ (Fig. 2c), we observe a linear dependence of *T*_c_ on *n*_2D_, i.e., *T*_c_ = 0.26 × 10^−13^ (K cm^2^)·*n*_2D_ – 0.1 K. Crucially, the coefficient for *n*_2D_ here is close to that found in the *n*_2D_ dependence of *T*_c_ obtained through chemical doping shown in Fig. 1c. The consistency between the electrostatic gating and chemical doping measurements highlights the predominant role of the carrier density, regardless of its origin, in tuning *T*_c_ at the KTO interface. Figure 2d shows that the critical current *I*_c_ also increases monotonically with *V*_G_. We note that in recent ionic liquid gating measurements^25^ on KTO, *T*_c_ also changes monotonically with *V*_G_, though the inferred *n*_2D_ does not.

### Enhanced *T*_BCS_ and a dome in *T*_BKT_ by electrostatic gating

**Fig. 3:**
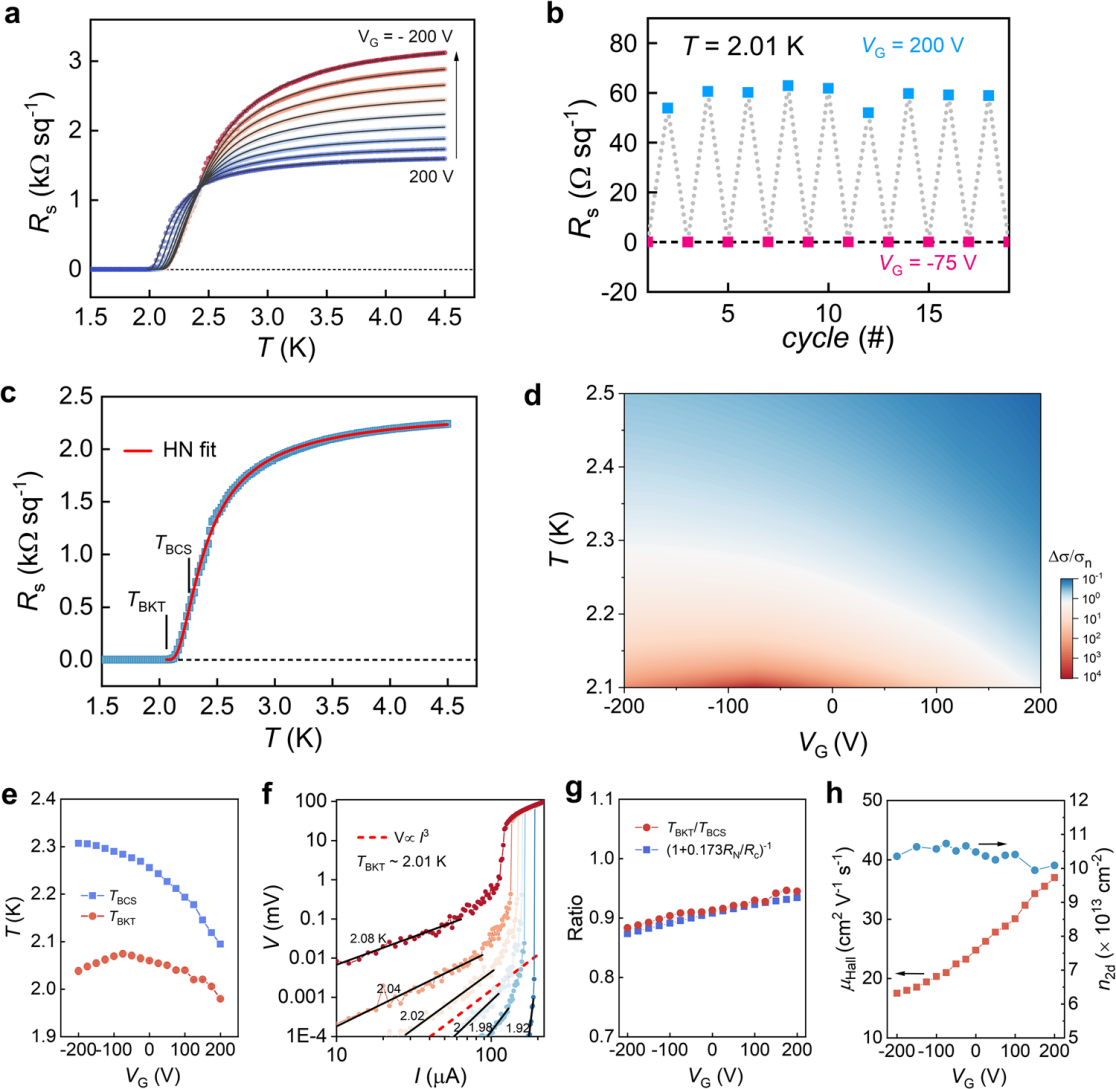
Electrostatic gating for a high *n*_2D_ sample. **a** Temperature dependence of *R*_s_ measured with different *V*_G_. The increment in *V*_G_ shown in the plot is 50 V. **b** Reversible switching of the superconductivity by *V*_G_ at *T* = 2.01 K. **c** Halperin-Nelson fit (solid line) to the *R*_s_(T) data. **d** Enhanced conductivity Δ*σ*/*σ*_n_ from superconducting fluctuations as a function of both *V*_G_ (*x*-axis) and temperature *T* (*y*-axis). **e** Mean-field (blue squares) and BKT (red dots) transition temperatures *T*_BCS_ and *T*_BKT_ as a function of *V*_G_. **f** Log-Log plot of the *V–I* measurement; the red dashed line is *V* *∝* *I*^*3*^, indicating *T*_BKT_ ~ 2.01 K. **g** Comparison of the ratio *T*_BKT_/*T*_BCS_ with theory. **h** Hall mobility (left axis) and carrier density (right axis) as a function of *V*_G_.

Next, we consider the effects of field-effect gating at higher carrier densities. Figure 3a shows *R*_s_(*T*) measured on KTO_9 with *n*_2D_ = 1.04 × 10^14^ cm^−2^, for different *V*_G_. As *V*_G_ varies from 200 V to – 200 V, *R*_N_ increases by about a factor of 2. At the same time, the superconducting transition occurs at higher temperatures, which is different than that seen on the low *n*_2D_ sample. Utilizing the response of superconductivity to *V*_G_, we show in Fig. 3b that the superconducting state with zero resistance can be switched on or off reversibly when *V*_G_ is varied between – 75 V and 200 V at *T* = 2.01 K. We note that a superconducting state is maintained at low temperature over the entire range of *V*_G_.

We found that the tunability of superconductivity here is due to an enhancement of the mean-field transition temperature *T*_BCS_ with negative *V*_G_. The transition from superconducting to normal state shown in Fig. 3a can be precisely interpreted by the Halperin-Nelson (HN) formula^26^, which describes the increase in resistance with rising temperature due to the Berezinskii-Kosterlitz-Thouless (BKT) transition arising from superconducting phase fluctuations in 2D, as well as amplitude fluctuations of Cooper pairs at still higher temperatures as proposed by Aslamasov and Larkin (AL). The solid curve in Fig. 3c (and all those in Fig. 3a) is a fit to the data using *R*_s_ (T) = *R*_N_(1 + 4 A^−2^sinh^2^(b(*T* – *T*_BKT_)^−1/2^*T*_BKT_^1/2^))^−1^, where A and b are fitting parameters that depend on the energy of the vortex core and the phase stiffness^27^. We find that the vortex core energy extracted from the fits is close to the expected value for an XY model, indicating that inhomogeneity does not need to be considered to get physical values for the fitting parameters (similar findings to ours, though with a lower value of the vortex core energy, have been recently reported^28^). In the HN expression, Δ*σ*/*σ*_N_ = 4A^−2^sinh^2^(b(*T*–*T*_BKT_)^−1/2^*T*_BKT_^1/2^) is a measure of the enhanced conductivity by superconducting fluctuations. Figure 3d shows a contour plot for Δ*σ*/*σ*_N_ as a function of *V*_G_ and temperature *T*, which is obtained from the HN analysis for measurements over the full range of *V*_G_. As *V*_G_ goes negative, Δ*σ*/*σ*_N_ shows monotonic enhancement for *T* ≥ 2.15 K, while at lower temperatures a local maximum in Δ*σ*/σ_N_ is seen.

From the HN analysis, we obtain the *T*_BKT_, which is found to be close to *T*_c0_, where *R*_s_ becomes zero. The mean-field *T*_BCS_ is obtained from the inflection point^29^ of the HN fit (maximum of its first derivative, near 20% of *R*_N_), and has been confirmed by AL fits (Supplementary Fig. 3). Figure 3e shows how *T*_BCS_ and *T*_BKT_ evolve as a function of *V*_G_. *T*_BCS_ is monotonically enhanced as *V*_G_ goes to negative values, while *T*_BKT_ shows a local maximum at *V*_G_ ~ –75 V. We also measured the voltage-current (*V*–*I*) characteristic near *T*_c0_ to determine *T*_BKT_ independently. As shown in Fig. 3f, *T*_BKT_ obtained through *V*-*I* measurements at *V*_G_ = 0 V is ~2.01 K, very close to the results obtained by the HN fit. See also Supplementary Fig. 4 for *V*–*I* measurements at different *V*_G_.

We find that the non-monotonic dependence of *T*_BKT_ on *V*_G_ is caused by the enhancement of *T*_BCS_ together with the increase of *R*_N_ as *V*_G_ decreases. The ratio of *T*_BKT_/*T*_BCS_ from our data, as shown in Fig. 3g, follows closely the prediction for a 2D superconductor^30^, i.e., *T*_BKT_/*T*_BCS_ = (1 + 0.173*R*_N_/*R*_c_)^−1^, where *R*_c_ = *ħ*/*e*^2^ with *ħ* being the reduced Planck constant. The increase of *R*_N_ by negative *V*_G_ is mainly due to charge carriers being pushed closer to the EuO/KTO interface, where more disorder is present^31,32^. As shown in Fig. 3h, the Hall mobility decreases from about 37 cm^2^ V^−1^ m^−1^ to 17 cm^2^ V^−1^ m^−1^ as *V*_G_ changes from 200 V to –200 V, while the variation in carrier density from Hall measurements is only ~5%. These observations show that, in addition to *n*_2D_ as found earlier, the proximity of the carriers to the interface also increases *T*_c_ (*T*_BCS_), suggesting the interface itself is the origin of pairing. We also observe that the critical field is enhanced at negative *V*_G_, which is found to be mainly due to the decrease in mobility (Supplementary Figure 5).

### Pairing mechanism at the KTO interfaces

We first discuss the marked proportionality of *T*_c_ with *n*_2D_ observed for the KTO (111) interface. One possible explanation for this phenomenon is that *T*_c_ is limited by the phase stiffness of the superconducting state, where *T*_c_ ∝ *E*_J_ (*T*_BKT_) with *E*_J_ = *ħ*^*2*^*n*_s_(*T*)/4 m^*^, n_s_ the 2D superfluid density, m* the effective mass of the electrons, and *n*_s_(0 K) ∝ *n*_2D_. This would give a linear relation between *T*_c_ and *n*_2D_. However, our analysis in Fig. 3c–h shows that *T*_BKT_ is close to *T*_BCS_, and in fact this is the case for samples over the full doping range studied. The closeness of *T*_BKT_ and *T*_BCS_ is expected for a conventional 2D superconductor, where the temperature range over which BKT physics is dominant is small, while the initial resistance drop is due to amplitude fluctuations of Cooper pairs that control the transport over most of the temperature range associated with the superconducting transition. Therefore, in these KTO samples, the transition is mainly governed by *T*_BCS_.

The above discussion leads to the second possibility for the observed proportionality between *T*_c_ and *n*_2D_, which is that the pairing interaction is non-BCS like with a superconducting gap Δ ∝ *E*_F_ (Fermi energy) instead of *ħ*ω_D_ (Debye energy). Such a situation would occur in the anti-adiabatic limit^33^ with $\hslash {\omega }_{D}$ > *E*_F_. Since in 2D, *n*_2D_ scales with *E*_F_, one would obtain *T*_c_ ∝ Δ ∝ *n*_2D_. The estimated *E*_F_ in our samples lies in the range of 10−80 meV, so pairing via the highest-energy longitudinal optical mode (LO4) with energy ~100 meV would be required. There are several potential issues with such a scenario. First, whether *ħ*ω_D_ can by replaced by *E*_F_ in the BCS expression for *T*_*c*_ is controversial^5^. Second, it would require that the BCS coupling constant be independent of *n*_2D_.

**Fig. 4:**
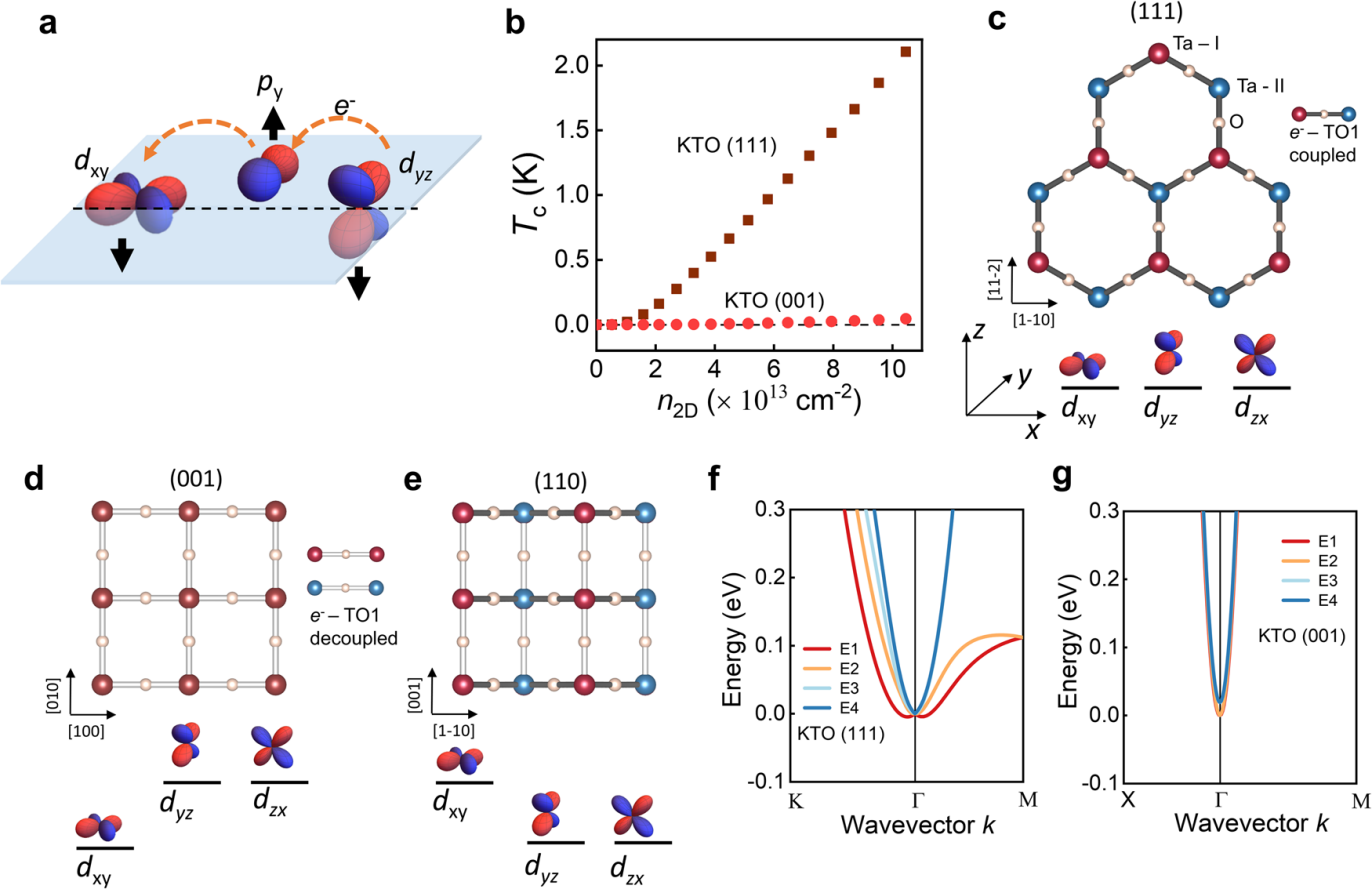
Pairing via transverse optical phonons. **a** Illustration of inter-orbital hopping of electrons between adjacent Ta sites via the O *p*_y_ orbital that becomes allowed by inversion symmetry breaking displacements (shown by the black arrows) of O and Ta atoms due to a TO1 phonon. Here, 3d orbital profiles are shown for illustration purposes. The 5d orbitals for Ta have the same angular symmetries but possess extra nodes in the radial direction. **b** Calculated *T*_c_ versus *n*_2D_ from TO1 phonon pairing for KTO (111) and (001) interfaces, the latter assuming *λ*_001_ = *λ*_111_/2. **c**–**e** Lattice structures formed by the first two layers of Ta and O atoms along the [111], [001], and [110] axes of KTO, respectively. Ta atoms in the upper and lower layers are shown in red and blue, respectively. The dark and light color of Ta-O-Ta bonds indicates the presence or absence of electron-TO1 phonon coupling, respectively. The degeneracy of the *d*_xy_, *d*_yz_, and *d*_zx_ orbitals in energy for each of these KTO surfaces is indicated below each panel by their vertical positions, the degeneracy at the lowest energy (3, 1, 2) correlating with *T*_c_. **f** Rashba-like splitting of bands for a KTO (111) bilayer due to displacements of Ta and O atoms perpendicular to the surface. **g** A similar displacement of atoms for the (001) surface does not produce a noticeable Rashba-like splitting.

This leads us to consider pairing via the soft transverse optical (TO1) phonon mode^34^, the phonon responsible for the nearly ferroelectric behavior of KTO. We note that the standard gradient coupling of the electrons to TO modes vanishes as the momentum transfer *q* goes to 0, meaning that only pairs of phonons can couple to the electrons^35^. However, as pointed out in recent work^36,37^, inversion breaking from the TO1 mode leads to linear coupling to electrons for inter-orbital interactions among the three *t*_2g_ orbitals: *d*_xy_, *d*_yz_, and *d*_zx_, which is illustrated in Fig. 4a. The displacements of Ta and O sites in the vertical direction, indicated by the black arrows, break inversion symmetry with respect to the horizontal plane joining the center of the *d* orbitals. As a result, the inter-orbital hopping amplitude of electrons along a Ta-O-Ta bond is no longer zero.

We show in detail in the Methods section that: (i) The TO1 mode energy (ω_ΤΟ1_) increases as a function of *n*_2D_ due to the electric field screening provided by the charge carriers. (ii) The BCS coupling constant *λ* (arising from the Rashba-like splitting of the energy bands with wavevector *k*) also increases with *n*_2D_. We take *T*_c_ = 1.14 ω_ΤΟ1_(*q* = 2*k*_F_, *n*_2D_) exp[–(1 + *λ*)/*λ*]. Adjusting *λ* = *c n*_2D_ < 1/ω_ΤΟ1_^2^ > by a free parameter *c* to scale the value of *T*_c_ to equal the experimental value of ~2 K at *n*_2D_ = 1 × 10^14^/cm^2^, we obtain a nearly linear dependence of *T*_c_ on *n*_2D_ for the KTO (111) interface, which is shown in Fig. 4b. Here, <> is a Fermi surface average (see Methods) and we assume that ω_ΤΟ1_^2^(*q* = 0) = *c*_1_ + *c*_2_ *n*_2D_ as observed in SrTiO_3_, with *c*_1_ and *c*_2_ being constants.

This scenario also predicts the absence of superconductivity for the KTO (001) interface, i.e., the superconductivity is highly sensitive to the crystallographic orientation. This is due to the nature of the inter-orbital electron-TO1 phonon coupling and the effects of quantum confinement for electrons at the KTO-oxide interface. The degeneracy of the *t*_2g_ orbitals for the 2DEG is lifted by quantum confinement depending on the crystalline surface normal, which is illustrated in Fig. 4c–e for the three KTO surface orientations. This lifting of degeneracy reduces the number of *d* orbitals participating in the inter-orbital hopping. Consequently, electrons and TO1 phonons are maximumly coupled for the KTO (111) surface where the three *t*_2g_ orbitals are degenerate, while they are largely decoupled for the KTO (001) surface given the splitting between the *d*_xy_ and *d*_xz/yz_ orbitals. Shown in Fig. 4f are the calculated energy bands for the KTO (111) surface exhibiting large ‘Rashba’-like splitting due to inter-orbital hopping, while the energy bands for the KTO (001) surface shown in Fig. 4g do not exhibit a noticeable splitting. Further, we find that the electron-TO1 phonon coupling strength for the KTO (110) surface is intermediate because the inversion breaking does not occur along the [001] crystal axis (Fig. 4e) in the (110) plane, reflecting the reduced orbital degeneracy of (110) relative to (111). We note that the apparent saturation of *T*_c_ for the KTO (110) interface may be due to its unusual electronic structure, with the predicted Fermi surface intersecting the zone boundary for *n*_2D_ ~ 7 × 10^13^ cm^−2^. This and other aspects of the (110) data will be presented in a future paper.

A more detailed analysis of pairing in KTO is highly non-trivial. It would also need to account for splitting of the TO1 mode due to both symmetry breaking and the electric field at the interface. As the inversion breaking is largest at the interface, this would be consistent with our observed increase of *T*_BCS_ when the charge carriers are pushed closer to the interface by a negative *V*_G_. A detailed analysis also needs to consider (1) the influence of both LO and TO modes on *T*_c_, (2) the carrier distribution *n*_3D_(*z*) along the interface normal (*z*) together with the strong interface orientation dependent electronic structure^38–40^, and (3) the influence of inter-orbital terms between the *t*_2g_ and *e*_g_ orbitals. We note that while the dependence of *T*_c_ on crystal orientation is not observed^9,41,42^ in SrTiO_3_ based 2DEGs (barring a recent exception^43^), this may be due to a weaker lifting^44–46^ of the degeneracy of the 3*d* orbitals (the confinement potential being weaker in STO than KTO). We also note that while the *t*_2g_ orbitals in bulk KTaO_3_ are degenerate, it is not known to superconduct^47^. We believe this may be due to the relatively low density of states in doped bulk KTO samples. In 3D, the density of states, *N*, is proportional to *k*_F_ whereas in 2D it is a constant. For the range of carrier densities we explore, *N*_3D_ is significantly smaller than *N*_2D_, leading via *λ* to an exponential suppression of *T*_*c*_ in the 3D case (see Methods section and Supplementary Fig. 6).

## Methods

### Material growth

The growth steps include annealing the substrate at a temperature ~600 °C, and then exposing it to a flux of Eu atoms in the temperature range 375−550 °C, both carried out for pressures in the 10^−10 ^Torr range. The annealing process promotes O vacancy formation near the surface, and subsequent exposure to Eu causes the formation of a thin layer of EuO_x_, as the Eu scavenges O from the substrate^19^. Following this step, the EuO overlayer is grown under a O_2_ partial pressure between 0.5–10 × 10^−^^9 ^Torr with a final EuO thickness of ~25 nm. Our microscopy results^19^ indicated the presence of O vacancies and Eu substitution on the K sites in KTO, which would both promote the formation of an interfacial electron gas. Upon varying the temperature at which the KTO substrate is exposed to Eu in vacuum, we were able to vary the doping levels in the 2DEG. We note that the EuO/KTO interface can show transport anisotropy or a ‘stripe’-like phase in the (111) plane^19^. However, this does not influence the analysis of *T*_c_, at which global superconductivity sets in as that is found to be the same for ‘stripe’ like samples at a given value of *n*_2D_. We also find that in samples with ‘stripe’ like behavior the anisotropy is strongly reduced when patterned into Hall bars vs those that are measured using a van der Pauw geometry, details of which are currently being studied. The samples presented here show weak or no anisotropy.

### Evolution of donor states as a function of doping

*n*_2D_ in these samples is determined using Hall measurements at 10 K. Supplementary Figure 1c shows the Hall resistance *R*_H_ measured as a function of magnetic field for different KTO (111) samples. Notably, the field dependences of *R*_H_ are all linear, with negative slopes, consistent with electron-like charge carriers. We found that the donor state of charge carriers evolves gradually from shallow to deeper energy as *n*_2D_ increases. Supplementary Figure 1d shows the temperature dependence of *n*_2D_ in the range 300 K–2 K. For samples with *n*_2D_(10 K) ≲ 5 × 10^13 ^cm^−2^, the measured *n*_2D_(*T*) increases upon cooling. This suggests that charge carriers in these samples mainly come from shallow donors whose energies merge with the conduction band due to the nearly 20-fold increase in the dielectric constant in KTO upon cooling. For samples with *n*_2D_(10 K) ≳ 5 × 10^13 ^cm^−2^, *n*_2D_(*T*) decreases upon cooling. This suggests that at higher doping levels charge carriers originate from deeper donor states^48^, to which some of them freeze out upon cooling. Supplementary Figure 1d also shows that regardless of the doping level in the as-grown samples, *n*_2D_ remains nearly constant for temperatures below 10 K for all samples.

### Scaling relation of coherence length

We have measured the Ginzburg-Landau coherence length (ξ_GL_) in the KTO (111) samples with different *n*_2D_, by measuring the out-of-plane upper critical field *B*_c2_ as a function of temperature. *B*_c2_ shows a linear temperature dependence near *T*_c_, from which we calculate ξ_GL_ using *B*_c2_(*T*) = Φ_0_(1–*T*/*T*_c_)/[2π(ξ_GL_)^2^], where Φ_0_ is the magnetic flux quantum. Supplementary Figure 2a shows the data of *B*_c2_ versus *T* for five samples. The *B*_c2_ varies greatly among these samples with different *n*_2D_ and *T*_c_. Supplementary Figure 2b summarize the ξ_GL_ for all the samples measured, which lies in the range from 10 to 57 nm. We found that *ξ*_GL_ scales with (μ/*T*_c_)^1/2^. As shown in Supplementary Fig. 2b, the ratio of *ξ*_GL_ to (μ/*T*_c_)^1/2^ remains constant for samples over the entire *n*_2D_ range. We found that this relation also holds when the superconductivity is tuned by *V*_G_ (see Supplementary Fig. 5).

For a BCS superconductor in the dirty limit, it is expected that *ξ*_GL_ ∝ (*l*ξ_BCS_)^1/2^ with ξ_BCS_ being the BCS coherence length^49^. Using *l* ∝ *v*_F_μ and ξ_BCS_ ∝ *v*_F_/*T*_c_ with *v*_F_ being the Fermi velocity, one gets *ξ*_GL_ ∝ *v*_F_(μ/*T*_c_)^1/2^. For the KTO (111) interface, we observe that *ξ*_GL_ ∝ (μ/*T*_c_) ^1/2^ instead. This would imply that *v*_F_ is a constant, independent of *n*_2D_. One way this could happen is if there is a linear dispersion of energy versus momentum like in graphene, though this conflicts with the known electronic structure of the KTO conduction bands that have a parabolic dispersion around the Γ point.

### Calculation of *T*_c_ versus *n*_2D_ from TO1 mode pairing

#### Evaluation of TO1 mode energy

The BCS formula for pairing from a TO1 mode is taken to be *T*_c_ = 1.14 ω_ΤΟ1_(*q* = 2*k*_F_, *n*_2D_) exp[–(1 + *λ*)/*λ*] where *λ* is the BCS coupling constant (any Coulomb pseudopotential, μ*, has been ignored). We first discuss the assumptions behind this expression, and then how to evaluate the various quantities. The first assumption is that the relevant pairing scale is controlled by the momentum transfer *q* of the TO1 mode for scattering around the Fermi surface, hence we set the cut-off energy to the maximum mode energy which is at *q* = 2*k*_F_. Therefore, the *n*_2D_ dependence of *T*_c_ will come from any hardening of the TO1 mode along with the increase of *k*_F_ with *n*_2D_ since the TO1 mode has a substantial dispersion. The TO1 mode dispersion is taken from the low energy neutron scattering study of Farhi et al.^50^. There, the phonon dispersion is fit with an expression due to Vaks, and in the approximation where the anisotropy of the dispersion is ignored involves solving a 2 by 2 matrix that couples the transverse acoustic mode to the transverse optic one. We do this using the parameters of Ref. ^50^. evaluated at their lowest temperature of measurement (10 K). To proceed further, we need to know the *n*_2D_ dependence of the mode energy at *q* = 0, as well as that of *k*_F_. For the former, we will assume that ω_ΤΟ1_^2^(*q* = 0, *n*_2D_) = *c*_1_ + *c*_2_ *n*_2D_; this relation is found in STO where doping of the bulk with free carriers has been observed^51^ (similar studies do not exist for bulk KTO). The resulting mode hardening is consistent with the electric field dependence of the Raman data of Fleury and Worlock^52^. *c*_1_ is set by the bulk value of ω_ΤΟ1_ of 2.5 meV. We set *c*_2_ by the estimated value of ω_ΤΟ1_ at *n*_2D_ = 10^14 ^cm^−2^ of 5.6 meV. The latter has been obtained from Ueno et al.^53^ that relates the field dependent dielectric function to *n*_2D_, with the relative dielectric function expressed as ε(*E*) = 4500/(1 + *b* *E*) with *b* = 8 × 10^−7^ (*E* in V m^−1^). The relevant “average” field *F* is then determined as in ref. ^54^. by assuming a triangular confining potential along *z* (the normal to the interface), and integrating the dielectric constant with respect to field up to *F*. That is, *e*·*n*_2D_ = 2 _0_∫^*F*^ε_0·_ε(*E*)d*E*, where *e* is elementary charge and ε_0_ is 8.85 pF m^−1^. The mode energy is then given by the Lyddane-Sachs-Teller relation ω_ΤΟ1_^2^(*q* = 0, *n*_2D_) = ω_ΤΟ1_^2^(*q* = 0, *n*_2D_ = 0) ε(0)/ε(*F*) that was found to be obeyed in the field dependent data of ref. ^51^. The reason we did not use this formalism over the entire range of *n*_2D_ is that the resulting *n*_2D_ dependence of ω_ΤΟ1_^2^(*q* = 0) deviates from the above assumed linear behavior (Supplementary Fig. 6e), which in turn has a detrimental impact on the functional dependence of *T*_c_ on *n*_2D_ (Supplementary Fig. 6f, where a dome is found instead). In the future, this could be looked at by measuring the actual TO1 mode energy as a function of *n*_2D_ in KTO (realizing that the TO1 mode polarized normal to the interface should harden more with *n*_2D_ than the TO1 mode orthogonal to this).

To obtain the variation of *k*_F_ with *n*_2D_, we assume the simple tight binding model of ref. ^55^, where the electronic structure is that of a (111) bilayer, with the parameters determined in order to reproduce the ARPES data from the (111) KTO surface of ref. ^40^. This results in a near neighbor hopping energy *t* of 1 eV, with the spin-orbit coupling ξ = 0.265 eV (in order to reproduce the quartet-doublet spin-orbit splitting of 0.4 eV at Γ). For *k*_F_, we take that of the larger of the two Fermi surfaces (the quartet splits into two doublets upon dispersing away from Γ), and set *k*_F_ to its value along Γ-K of the surface Brillouin zone. *n*_2D_ is gotten by determining the occupied area of the two Fermi surfaces at *E*_F_. The resulting dependence of *E*_F_ and the TO1 mode energies with *n*_2D_ is shown in Supplementary Fig. 6a. One can see the approximate linear dependence of *E*_F_ with *n*_2D_ (expected for a parabolic dispersion in 2D), with the deviation at larger *n*_2D_ due to the approach to a van Hove singularity of the lower band of the quartet that occurs at the M point of the surface zone. As one can also see, the dependence of the mode energy at *q* = 2*k*_F_ is not linear in *n*_2D_. This is to be expected, since in the approximation where the mode energy at *q* = 0 is small compared to that at *q* = 2*k*_F_, the mode energy should scale with *q* and thus *k*_F_, the latter going approximately as the square root of *n*_2D_. Also note that over most of the *n*_*2D*_ range, the mode energy at *q* = 2*k*_F_ is much smaller than *E*_F_, justifying the BCS (adiabatic) approximation for TO1 mode pairing. We note that although our experimental results, such as the orientational and gate-field dependences of superconductivity, suggest an underlying 2D electronic structure, we cannot rule out the existence of ‘3D’ electronic states at KTO (111) interfaces.

### BCS coupling constant *λ*

For TO1 mode pairing, *λ* is given by:^37^ *λ* = *c n*_2D_ < 1/ω_ΤΟ1_^2^(*q*, *n*_2D_)> where <>  represents an average over the Fermi surface. As the mode dispersion is quadratic in *q*, the Fermi surface average is trivial, resulting in *λ* = *c*·*n*_2D_/[ω_ΤΟ1_(*q* = 2*k*_F_, *n*_2D_)·ω_ΤΟ1_(*q* = 0, *n*_2D_)]. Here, *n*_2D_ in the numerator comes from *k*_F_^2^ (*k*_F_^2^ = 2π·*n*_2D_). This factor arises from the electron-phonon vertex that is proportional to *k*_F_ (that is, the effect of inversion breaking from the polar TO1 mode leads to a linear splitting of the energy bands with *k*). More will be described about this inversion breaking effect in the next section. The constant *c* subsumes the proportionality coefficient of the vertex along with the density of states at the Fermi energy, *N*_F_, that we assume is constant (equivalent to linearity of *E*_F_ with *n*_2D_). This is in contrast to the bulk^56^ where *N*_F_ goes as *n*_3D_^1/3^. The value of *c* would require a detailed microscopic theory. In lieu of that, we set *c* in order to give *T*_c_ of ~2 K at *n*_2D_ = 10^14^ cm^−2^ (for this *n*_2D_, *λ* ~ 0.26 and so *c* = 2.85 when *n*_2D_ is in units of 10^13^ cm^−2^ and ω_TO1_ in meV). The resulting *λ* and *T*_c_ versus *n*_2D_ is plotted in Supplementary Fig 6b, c, respectively. *T*_c_ is then replotted in Supplementary Fig 6d assuming that *λ* is reduced by a factor of 2.

One can see a remarkable linear variation of *T*_c_ with *n*_2D_ in Supplementary Fig. 6c, despite the non-linearity with *n*_2D_ of both the BCS cut-off and the BCS coupling constant. We note that the variation of *T*_c_ with *n*_2D_ is a result of the interplay between the dependences of ω_TO1_ and the electron-phonon coupling *λ* on *n*_2D_. Obtaining a linear behavior of *T*_c_ is sensitive to details of both dependences. However, the rise in *T*_c_ with *n*_2D_ is a general finding, in part due to the increase of ω_TO1_ with *n*_2D_ along with the strong dispersion of the mode. Therefore, we expect this rise will cease once the phonon wavevectors spanning the Fermi surface approach the zone boundary.

In Supplementary Fig. 6d, one sees a strong suppression of *T*_c_, with values similar to those claimed by Ueno *et al*. for the (001) interface^53^ (a further reduction in *λ* would completely suppress *T*_c_ as we observe). Since *λ* is proportional to the square of the electron-phonon vertex, a mere reduction of the vertex by 2^1/2^ is sufficient to cause an enormous reduction in *T*_c_. As such, we expect *T*_c_ to be extremely sensitive to the interface orientation, which we address next. We note that in a more recent work^25^ using ionic liquid gating of the KTO (001) surface, superconductivity was not found.

### Dependence of *T*_c_ on interface orientation from TO1 mode pairing

#### Electron-phonon vertex

To obtain a significant electron-phonon vertex for a TO1 mode, one requires inter-orbital terms (intra-orbital ones instead lead to a coupling quadratic in the ion displacement^35^). Therefore, in a TO1 mode picture, one requires orbital degeneracy in order to get a substantial *T*_c_. This qualitatively explains the observed dependence of *T*_c_ with interface orientation. For the (111) case (ignoring any trigonal distortion), the three *t*_2g_ states are degenerate at Γ (being split into a lower quartet and an upper doublet by spin-orbit). We can contrast this with the (001) case, where confinement along [001] leads to the *d*_xy_ state being pulled down substantially relative to the *d*_xz/yz_ ones. This splitting acts to suppress the effect of inter-orbital terms, consistent with our lack of observation of superconductivity on (001) interfaces (in ref. ^53^, a *T*_c_ of <50 mK was observed). For (110), we expect something intermediate, because the effect of confinement in that case is to instead pull down the *d*_xz/yz_ states relative to the *d*_xy_ one (so, partial degeneracy).

To go beyond these qualitative observations, we need to consider the variation of the electronic structure with interface orientation, and we also need an estimate of the coupling of these electrons to the TO1 mode. To address the latter, we follow ref. ^37^, where the TO1 mode is considered to be a Slater mode (known to be a good approximation for the TO1 eigenvector). As they show, there are two contributions. One is the standard gradient one which only involves the metal ion motion and disappears as *q* goes to 0. The second is the inter-orbital terms that involves primarily the oxygen ion motion and does not vanish as *q* goes to 0.

The inversion splitting of the bands near Γ due to the TO1 mode gives rise to an electron-phonon vertex^37^ g_TO1_ = 2*t*ʹ*c*ʹ*dk*_F_*a* where *t*ʹ is the derivative of the near-neighbor hopping with respect to the ion displacement, *c*ʹ = (*η*^−1/2^ + *η*^1/2^) where *η* = 3*M*_O_/*M*_Ta_, *d* is the zero-point displacement of the ions from excitation of the Slater mode given by *d* = (ℏ/2*M*_S_ω_TO1_)^1/2^, *M*_S_ is the mass of the Slater mode (3*M*_O_ + *M*_Ta_), and *a* is the lattice constant (3.9884 Å for KTO). The existence of *t*ʹ allows coupling between one *t*_2g_ orbital on a given Ta site with a different *t*_2g_ orbital on a neighboring Ta site (which is not allowed if the Ta-O-Ta bond is linear). *t*ʹ was obtained in ref. ^37^. from a frozen phonon calculation. Instead, we follow Ref. ^36^. which calculates the inter-orbital coupling due to displacement of the oxygen ion transverse to the Ta-O-Ta bond. This leads to a value of *t*ʹ = 2*t*_pd_^2^/(Δ_pd_*a*) where *t*_pd_ is the *pd*π hopping and Δ_pd_ the energy difference between the O 2*p* and Ta 5*d* orbitals, noting that the oxygen ion is at a distance *a*/2 relative to the Ta ion. *t*_pd_ and Δ_pd_ can be obtained from Mattheiss’ Slater-Koster fits^57^ for KTO. Doing this we obtain the values listed in Supplementary Table 1. *t*_R_ is the inversion breaking hopping parameter that enters the electronic structure Hamiltonian^55^, and the TO1 mode energy listed is that of the bulk insulator at *q* = 0. The reason we included STO is to compare our crude approximation of *t*ʹ to the more sophisticated one of Ref. ^37^. Our value for STO is about 50% larger than theirs (our value for KTO being similar to theirs for STO). The reason the STO value is larger is because of the softer TO1 mode coupled with the larger *d* (zero-point motion) due to the lighter Ti ion.

On the other hand, there are other inversion breaking terms that have been ignored here, and those couple the *t*_2g_ electrons to the *e*_g_ ones^58^. As those terms are proportional to the inversion breaking times the spin-orbit coupling divided by the *t*_2g_-*e*_g_ splitting, they are presumably much larger in KTO than STO given its 20 times larger spin-orbit coupling ξ. These terms also exhibit orbital differentiation. For instance, for displacements along [001], the *d*_*xy*_ states do not couple to the *e*_g_ states, and therefore similar considerations to those above apply for these *t*_2g_-*e*_g_ terms as well. These terms in turn could explain why *T*_c_ in KTO is much higher than in STO. But their inclusion results in a much more involved theory than that presented here, so we leave this to future work. In this context, note that our dispersion plots are based on displacements along the interface normal. In the dynamic case relevant for the calculation of *T*_c_, one or both TO1 modes (or neither) come into play depending on the specific orbitals and interface orientation involved.

Regardless, the purpose of this exercise was to give a rough estimate of the size of *t*_R_ due to dynamic coupling of the electrons to the TO1 mode. Its value above (~32.5 meV) is extremely large, as commented on in ref. ^37^, meaning that TO1 mode pairing is quite viable. In Fig. 4f, g and Supplementary Fig. 7a, we show the electronic structure in the bilayer approximation for the three interface orientations assuming this value of *t*_R_.

### Splitting in the energy dispersion and interface orientation variation of *T*_c_

For KTO (111) (Fig. 4f), note the pronounced Rashba-like effect near Γ (displaced parabola) for the lowest band of the spin-orbit quartet. For (001) (Fig. 4g), note the small effect of inversion breaking. For (110) (Supplementary Figure 7a), note the small splitting along Γ-Z (similar to (001)) and the much larger splitting along Γ-M, along [1-10]. This (110) case leads to a large Fermi surface anisotropy, while the (001) case is isotropic and the (111) case only mildly anisotropic, since the Ta-O-Ta bonds are parallel to [001] but are not parallel to [1-10] in the (110) plane. These electron-TO1 mode couplings for different KTO surfaces are schematically illustrated in Fig. 4c–e of the main text using dark and light colors for the Ta-O-Ta bonds.

We now address the interface orientation variation of *T*_c_ from the variation of the inversion breaking effect with orientation. Note that the *k*_F_^2^ factor in the numerator of the BCS coupling constant *λ* comes from the square of the splitting of the bands due to inversion breaking, the latter going like α_R_*k*_F_, with the notation α_R_ denoting that many (but not all) of these inter-orbital terms have a Rashba-like form. So, *λ* scales as α_R_^2^. α_R_ is equal to a constant times *t*_R_. We denote this constant by δ (which has units of Angstroms). So, *λ* scales as δ^2^. In Supplementary Table 2 are values of δ gotten from the slope of the lowest energy band versus *t*_R_ divided by |*k* | (*k* is taken to be near Γ), which we extract from that of the lowest band. An example is shown in Supplementary Fig. 7b.

The first thing to note from Supplementary Table 2 is that (001) will have a negligible *λ*. In fact, we can get an analytic estimate of what the δ ratio is from Ref. ^58^. The inversion breaking term for (111) is proportional to 3^1/2^ (ignoring coupling to *e*_g_ states). The inversion breaking for (001) is proportional instead to 2ξ/ε where ε is the splitting of the *d*_xy_ state from *d*_xz/yz_. Therefore, the ratio of δ (111 to 001) is 3^1/2^ε/2ξ. In our simple model, ε is equal to *t*. For our values (*t* = 1 eV, ξ = 0.265 eV), we get a ratio of 3.27, which approximately matches the numerical value from Supplementary Table 2 (3.78). Since *λ* goes as the square of this ratio, one sees that *T*_c_ on the (001) interface should be negligible.

For (110), it is difficult to give an estimate given the large anisotropy of the inversion breaking in the surface Brillouin zone coupled with the large anisotropy of the Fermi surface (the Fermi surface is predicted to be strongly elongated along [1-10]). Still the (110) δ values are much closer to the (111) value than the (001) value, so a *T*_c_ for (110) half that for (111) is reasonable. The upshot is that the *T*_c_ variation follows the breaking of orbital degeneracy, as shown by the last two columns of Supplementary Table 2. Here, “orbitals” refer to the *t*_2g_ orbitals present in the low energy manifold of states.

## Supplementary information

Supplementary Information

## Data Availability

All data presented here in the paper are available via the Harvard Dataverse at 10.7910/DVN/TKTVK1.
