# Supplementary material for: Tunable superconductivity and its origin at KTaO3 interfaces
Source: Nat Commun. 2023 Feb 20;14:951. doi: 10.1038/s41467-023-36309-2 (PMC9941122; doi:10.1038/s41467-023-36309-2)
Supplement: Supplementary file 1 — Supplementary Information [file 41467_2023_36309_MOESM1_ESM.pdf]

Supplementary information

# Tunable superconductivity and its origin at $KTaO_3$ interfaces

Changjiang Liu[1,2*], Xianjing Zhou[3], Deshun Hong[1], Brandon Fisher[3], Hong Zheng[1], John Pearson[1], Jidong Samuel Jiang[1], Dafei Jin[3], Michael R Norman[1*], Anand Bhattacharya[1*]

[1]Materials Science Division, Argonne National Laboratory, Lemont, IL 60439, USA.
[2]Department of Physics, University at Buffalo, SUNY, Buffalo, NY 14260, USA.
[3]Center for Nanoscale Materials, Argonne National Laboratory, Lemont, IL 60439, USA.

*Email: changjia@buffalo.edu; norman@anl.gov; anand@anl.gov*

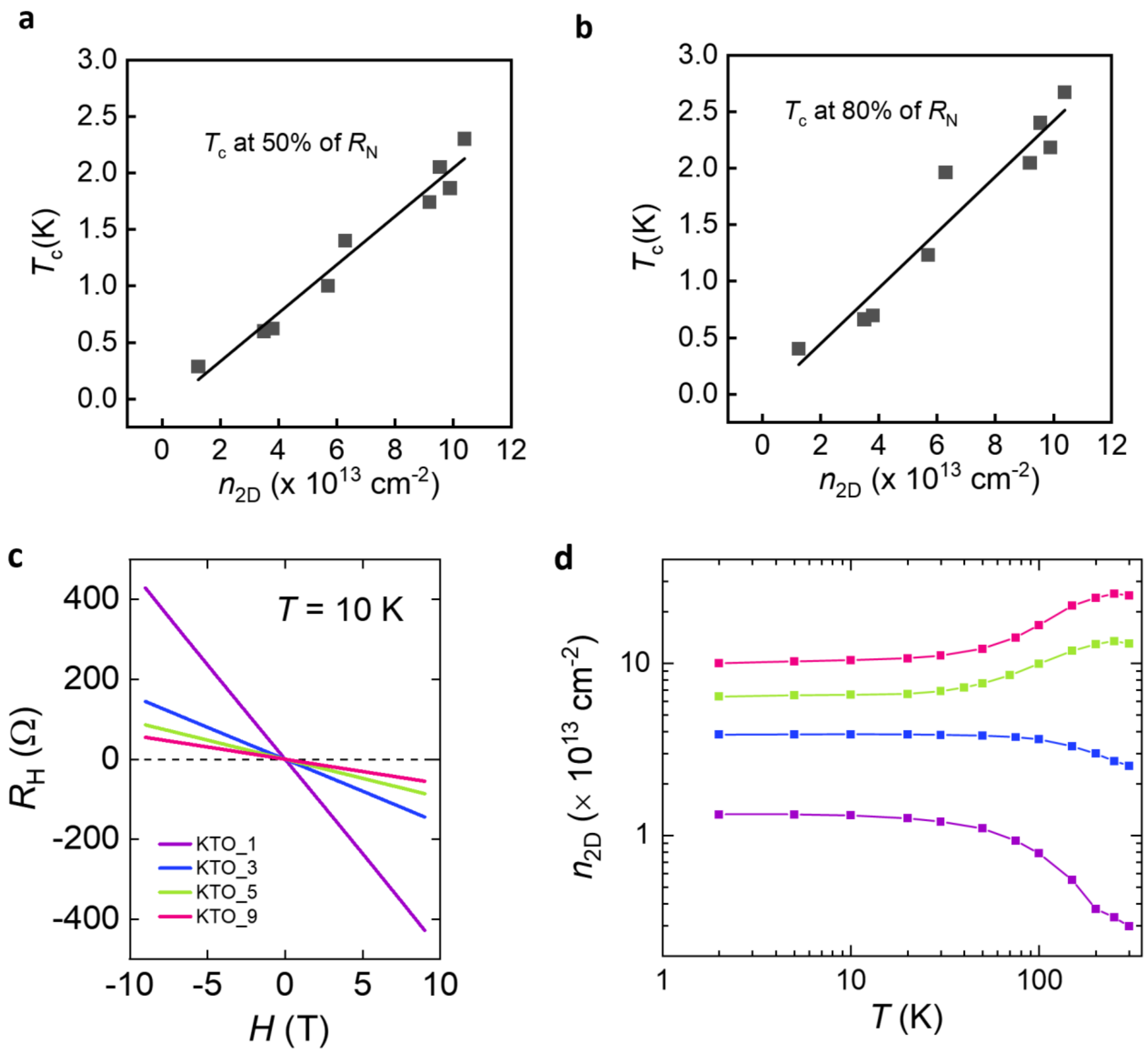


**Supplementary Figure 1. Linear relation between $T_c$ and $n_{2D}$ and the evolution of donor states for samples with different doping levels. a, b** $n_{2D}$ dependence of $T_c$ for $T_c$ determined by 50% and 80% of the normal state resistance, respectively. In both cases, a linear relation between $T_c$ and $n_{2D}$ can be observed. **c** Hall resistance as a function of magnetic field for four EuO/KTO (111) samples. **d** Temperature dependence of $n_{2D}$ in samples with different doping levels.

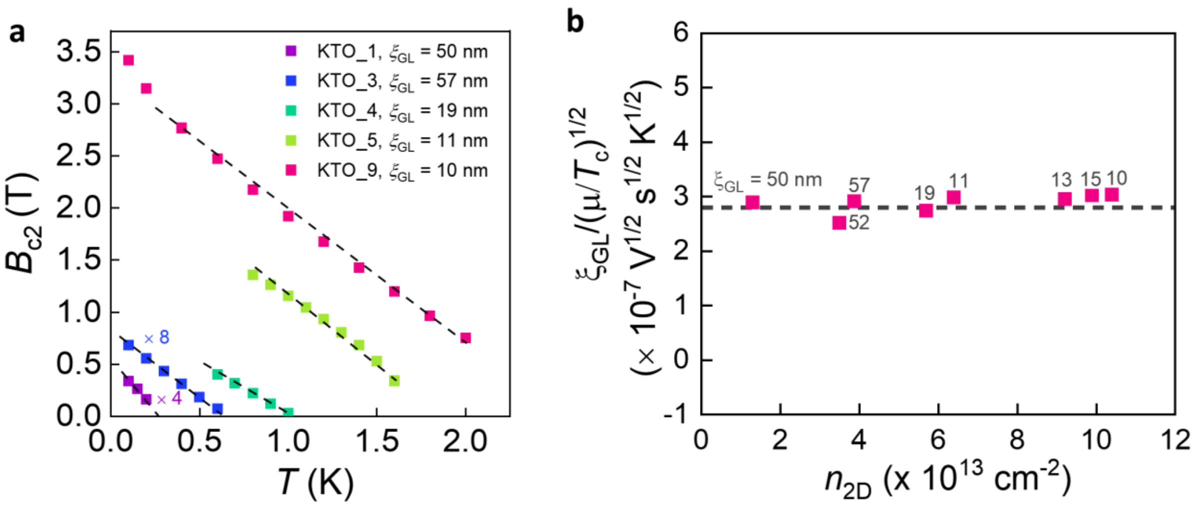


**Supplementary Figure 2. Coherence length measurements. a** Temperature dependence of the upper critical field measured for EuO/KTO(111) samples with different $n_{2D}$. **b** Scaling of the coherence length $\xi_{GL}$ obtained for different samples. The value of $\xi_{GL}$ is indicated by the number with units of nanometers.

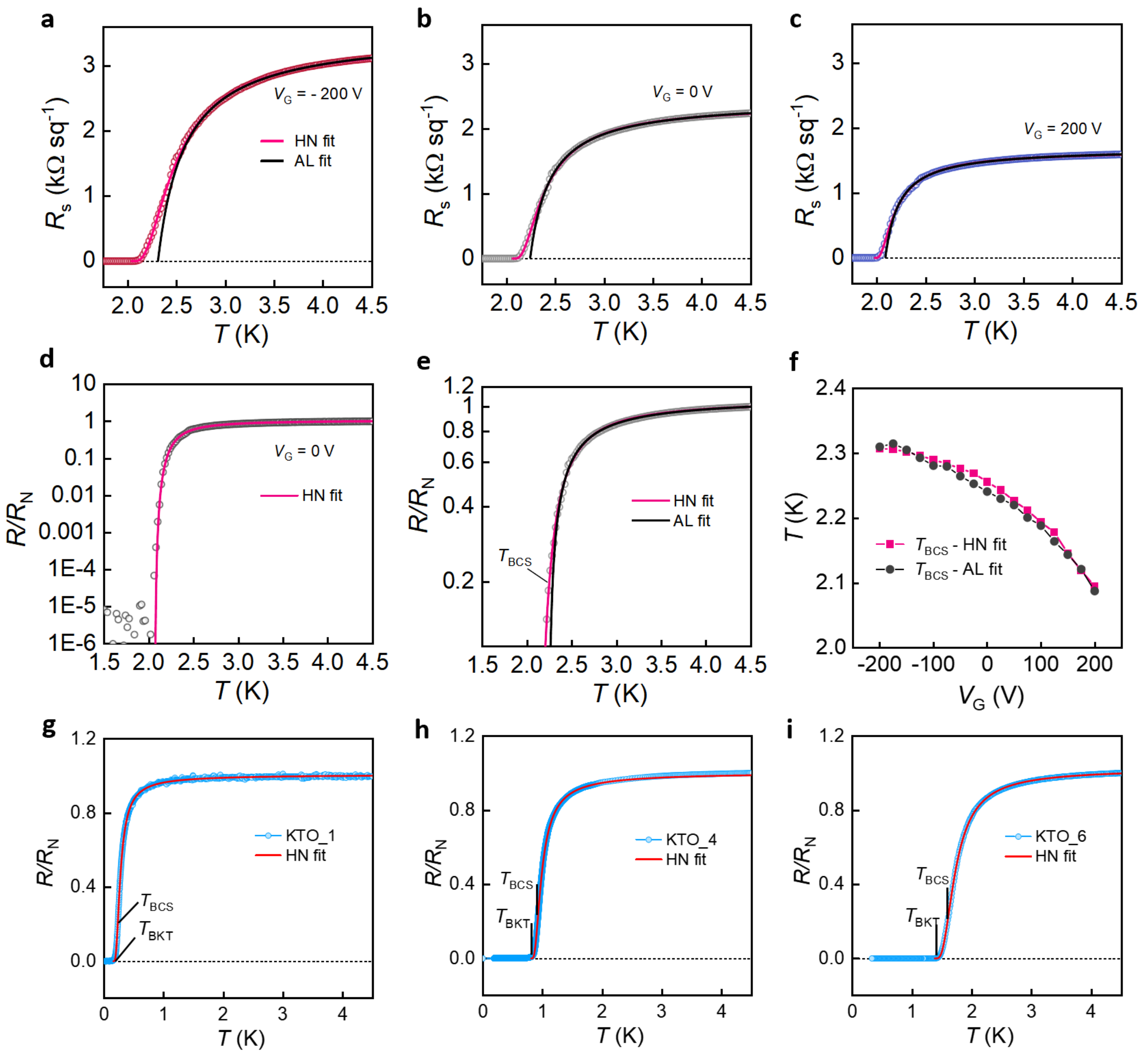


**Supplementary Figure 3. Comparison between the HN and AL fit and HN fit for samples with different doping. a – c** Red and black solid lines are the HN and AL fits to the data for three different $V_G$, respectively. At higher temperatures the two fitted curves are identical to each other. **d, e** A log-linear plot for the HN fit and its comparison with the AL fit, respectively. **f** The $T_{BCS}$ obtained from the HN and AL fits for different $V_G$ are shown in red and black, respectively. $T_{BCS}$ obtained from both methods show about the same $V_G$ dependence. **g – i** HN fit for KTO_1, KTO_4 and KTO_6, respectively. For all samples, the transition from the normal state into the superconducting state can be well described by the HN formula, which also shows that $T_{BKT}$ is close to $T_{BCS}$, with fluctuations associated with the latter dominating the transport over a large temperature range. The AL fit uses a formula $R$s = 1/((1/$R_N$) + (1/$R_c$)$T_{BCS}$/(T - $T_{BCS}$)), where $R_N$

and $R_c$ are fitting parameters. $R_N$ is determined by the normal state resistance of the sample. We note that $R_c$ should be equal to $16\hbar/e^2$ according to the AL formulation. However, we found that the obtained $R_c$ from fitting to the data is less than $16\hbar/e^2$ by about a factor of 2. The reasons for this discrepancy are not known at this time, though processes for conductance enhancement beyond AL (e.g. Maki-Thompson, density of states effects) may play a role.

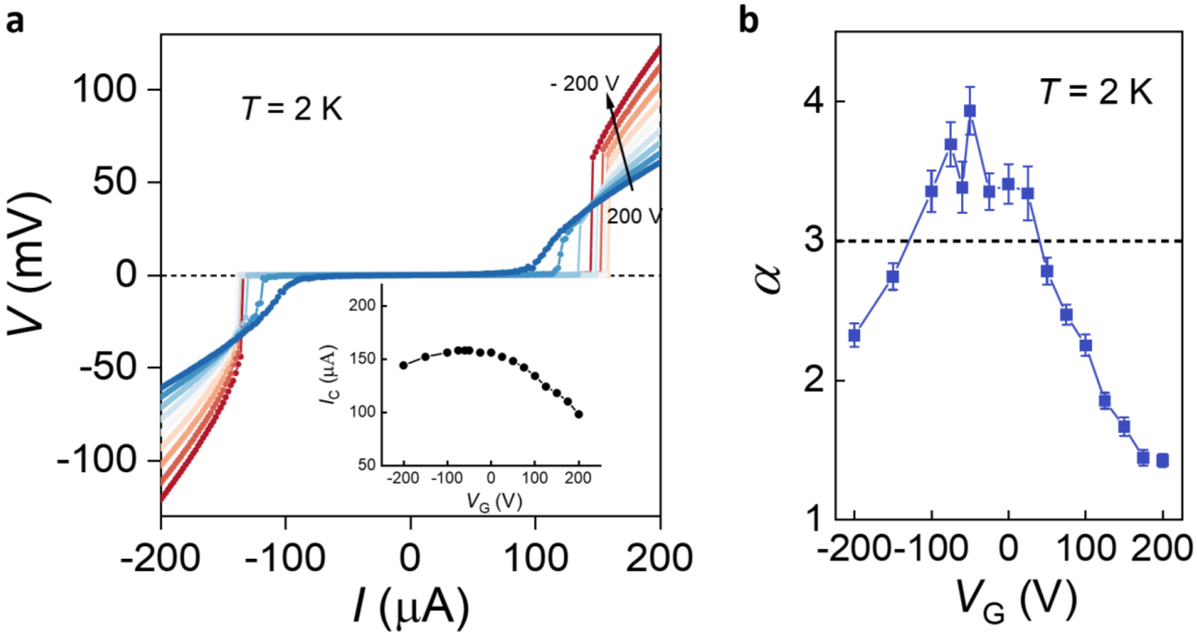


**Supplementary Figure 4. Critical current measurement for different $V_G$. a** $V - I$ measurements on KTO_9 at $T$ = 2 K under different $V_G$. The increment of $V_G$ showing in the plot is 50 V. Inset shows the critical current $I_c$ as a function of $V_G$. **b** Exponent $\alpha$ in $V \propto I^{\alpha}$ as a function of $V_G$. These $V - I$ measurements confirm that the $T_{BKT}$ is tuned by $V_G$. As shown in (**b**), the exponent $\alpha$ in $V \propto I^{\alpha}$ obtained in these $V - I$ measurements evolves as a function of $V_G$, showing a local maximum on the negative side of $V_G$. The horizontal dashed line is $\alpha = 3$, which indicates a BKT transition. A value of $\alpha$ that is greater than 3 suggests that the corresponding $T_{BKT} > 2$ K, while $\alpha < 3$ means $T_{BKT} < 2$ K. These $V - I$ measurement results obtained at a fixed temperature for BKT transition are qualitatively the same as those from the HN fit.

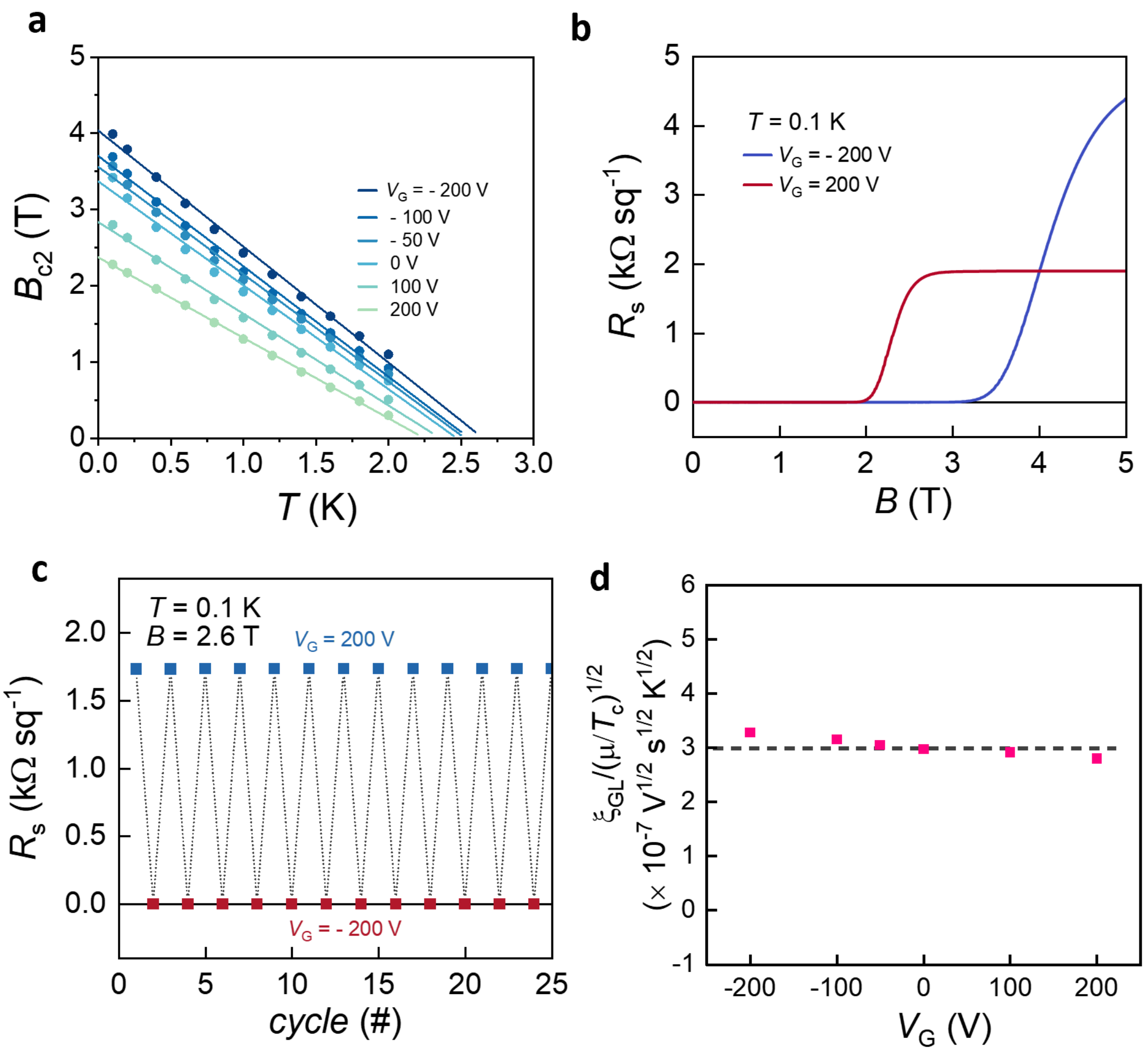


**Supplementary Figure 5. Tuning of critical field and SC on KTO_9 by $V_G$. a** Temperature dependence of the upper critical field measured at different $V_G$. **b** $R_s$ versus $B$ for two different $V_G$ at $T$ = 0.1 K. **c** Switching the SC by $V_G$ at $B$ = 2.6 T. Here the critical field at 0 K increases by about a factor of two as seen in (**a**), which is mainly due to the decrease in mobility from about 37 cm$^2$ V$^{-1}$ m$^{-1}$ to 17 cm$^2$ V$^{-1}$ m$^{-1}$ as $V_G$ changes from 200 V to – 200 V. This is in agreement with the scaling relation $\xi_{GL} \propto (\mu/T_c)^{1/2}$ discussed in the Methods section, since $\xi_{GL} \propto 1/B_{c2}^{1/2}$ and the variation in $T_c$ is only ~ 10%. Thus, the tunability of SC shown in (**c**) is a result of the strong dependence of the critical field on mobility. **d** The value of $\xi_{GL}$ / $(\mu/T_c)^{1/2}$ in the full range of $V_G$ is around 3 × $10^{-7}$ V$^{1/2}$ s$^{1/2}$ K$^{1/2}$ which is the same as that shown in Supplementary Fig. 2b.

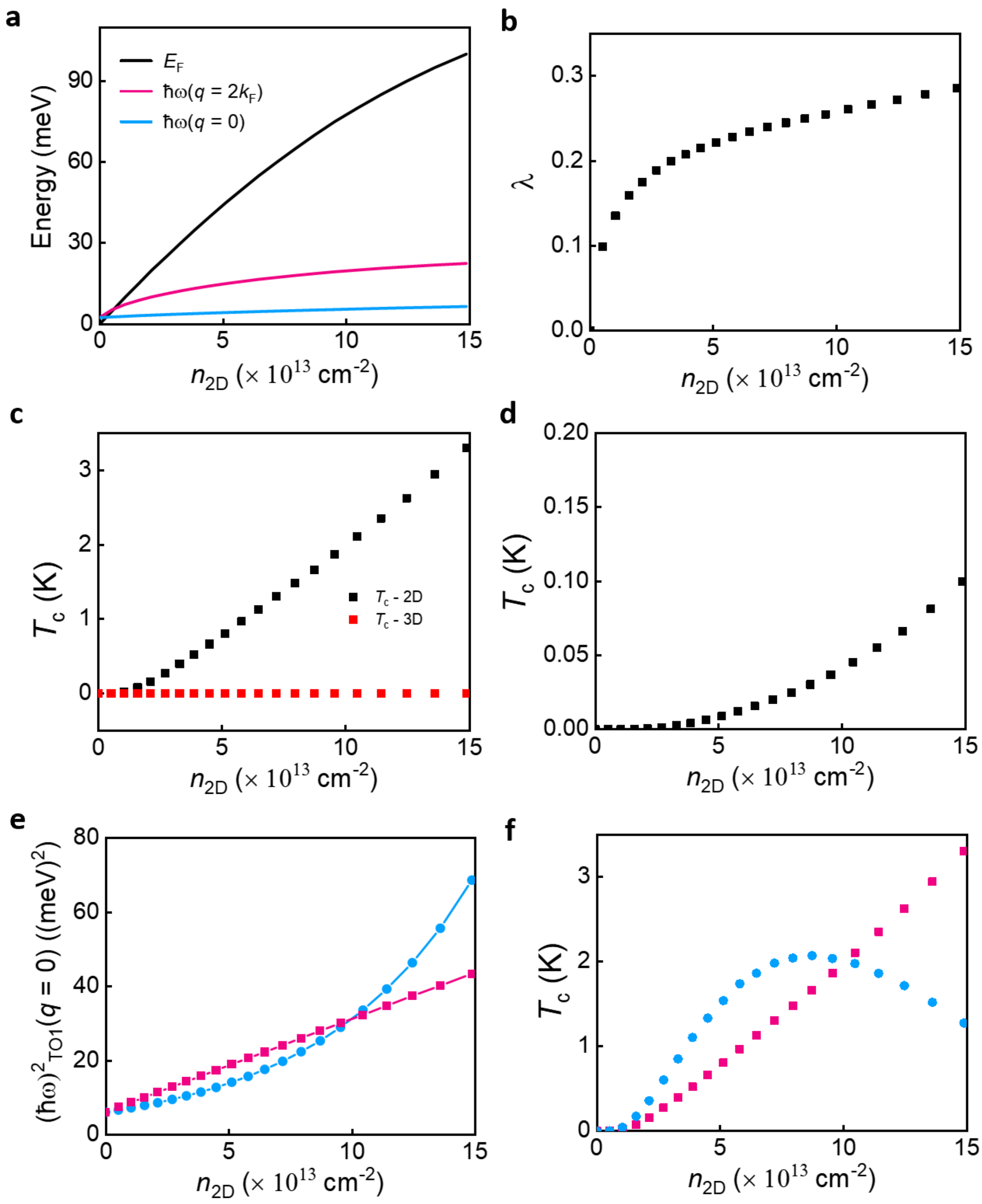


**Supplementary Figure 6. Calculation of TO1 mode energy, BCS coupling constant and $T_c$. a** Variation versus carrier density for the Fermi energy and TO1 mode energies. **b** BCS coupling constant as a function of $n_{2D}$. **c** Calculation of $T_c$ versus $n_{2D}$ with the prefactor of the coupling constant $c$ set to match the experimental $T_c$ value of 2 K at $n_{2D}$ = $10^{14}$ cm$^{-2}$. For comparison,

assuming the same $k_F$, the $T_c$ for the 3D case is found to be vanishingly small in comparison to the 2D case due to the reduced density of states in 3D in this carrier density range. **d** Calculation of $T_c$ versus $n_{2D}$ with the coupling constant set to half of that in (**c**). **e** Contrasting dependence of the TO1 mode energy at $q = 0$ on $n_{2D}$ from either a linear assumption (red) or that derived from a triangular potential approximation (blue). **f** Differing dependence of $T_c$ on $n_{2D}$ from (**e**). The red points in (**f**) are equivalent to those plotted in (**c**).

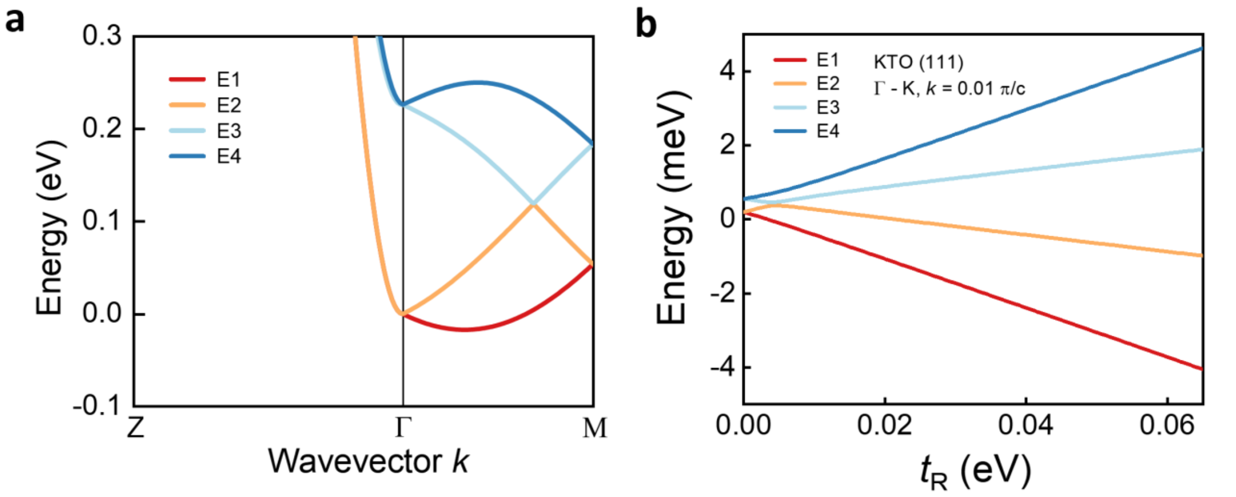


**Supplementary Figure 7. Calculation of the energy dispersion and splitting. a** Dispersion along symmetry lines of the surface Brillouin zone for a (110) orientation with $t_R$ = 32.5 meV. **b** Variation of splitting of the lowest lying quartet versus $t_R$ along Γ-K for (111), with $c = (2/3)^{1/2}a$. For (110), Z is along [001] and M along [1-10]. Results analogous to (**a**) for (111) and (001) are shown in Fig. 4f and 4g of the main text, respectively . $t_R$ is the inversion breaking term entering into the tight binding Hamiltonian.

| | $t_{pd}$ | $\Delta_{pd}$ | $t_{pd}^2/\Delta_{pd}$ | $\eta$ | $\omega_{TO1}$ | $d$ | $g_{TO}/k_F a$ | $t_R$ |
|---|---|---|---|---|---|---|---|---|
| **STO** | 1.136 | 4.018 | 0.321 | 1.003 | 0.0010 | 0.147 | 0.0968 | 0.0484 |
| **KTO** | 1.395 | 4.450 | 0.437 | 0.265 | 0.0025 | 0.060 | 0.0648 | 0.0324 |

**Supplementary Table 1. Calculation of parameters associated with TO1 phonon pairing.** $t_{pd}$ is the $pd\pi$ hopping, $\Delta_{pd}$ the energy splitting of the Ta *d* and O *p* orbitals, $\eta$ is the ion mass ratio $3M_O/M_{Ta}$, $\omega_{TO1}$ is the bulk TO1 mode energy at $q = 0$, *d* is the zero-point displacement of the O ions due to the TO1 mode, $g_{TO}$ is the electron-TO1 phonon vertex, and $t_R$ is the inversion breaking term entering into the tight binding Hamiltonian. Here, $k_F$ is the Fermi wavevector and *a* the bulk lattice constant. Units are eV and Angstroms.

| surface | direction | $\delta/a$ | $\delta_A/a$ | orbitals | $T_c$ |
|---|---|---|---|---|---|
| **(111)** | Γ-K | 1.712 | $3^{1/2}$ | xy, xz, yz | ~ 2 K |
| | Γ-M | 1.716 | $3^{1/2}$ | | |
| **(001)** | Γ-X | 0.453 | $2\xi/\varepsilon$ | xy | ~ 0 K |
| **(110)** | Γ-Z | 0.000 | 0 | xz, yz | ~ 1 K |
| | Γ-M | 1.414 | $2^{1/2}$ | | |

**Supplementary Table 2. Comparison of inversion splitting for the three crystallographic orientations.** $\delta$ for $k$ near Γ, with the inversion dependence of the lowest band energy going as $-\delta t_R k$, with $\delta_A$ a simple analytic estimate in the limit that $k$ goes to 0. "orbitals" denote those $t_{2g}$ orbitals present in the lowest energy bands. $T_c$ is the maximally observed $T_c$ for a given interface orientation. Here, a is the bulk lattice constant, $\xi$ the spin-orbit coupling, and $\varepsilon$ the energy splitting between the xy and xz/yz states.
